# Reduced expression of cenp-e in human hepatocellular carcinoma

**DOI:** 10.1186/1756-9966-28-156

**Published:** 2009-12-18

**Authors:** Zijie Liu, Kang Ling, Xia Wu, Ju Cao, Bin Liu, Suyan Li, Qiong Si, Yan Cai, Chen Yan, Yan Zhang, Yaguang Weng

**Affiliations:** 1 The key laboratory of laboratory medical diagnostics, ministry of education; the faculty of laboratory medicine, Chongqing Medical University, Chongqing PR China; 2 Department of surgery, the first affiliated hospital, Chongqing medical university, Chongqing, PR China; 3 Zhaotong Health School, Yunnan, PR China

## Abstract

**Background:**

CENP-E, one of spindle checkpoint proteins, plays a crucial role in the function of spindle checkpoint. Once CENP-E expression was interrupted, the chromosomes can not separate procedurally, and may result in aneuploidy which is a hallmark of most solid cancers, such as hepatocellular carcinoma (HCC). We investigate the expression of CENP-E in human hepatocellular carcinoma,. and analyze the effect of low CENP-E expression on chromosome separation in normal liver cell line (LO2).

**Methods:**

We determined its levels in HCC and para-cancerous tissues, human hepatocellular carcinoma-derived cell line (HepG2) and LO2 cell line using real time quantitative PCR (QPCR) and Western blot. Further to know whether reduction in CENP-E expression impairs chromosomes separation in LO2 cells. we knocked down CENP-E using shRNA expressing vector and then count the aneuploid in LO2 cells using chromosomal counts assay.

**Results:**

We found that both CENP-E mRNA and protein levels were significantly reduced in HCC tissues and HepG2 cells compared with para-cancerous tissues and LO2 cells, respectively. A significantly-increased proportion of aneuploid in these down-knocked LO2 cells compared with those treated with control shRNA vector.

**Conclusions:**

Together with other results, these results reveal that CENP-E expression was reduced in human HCC tissue, and low CENP-E expression result in aneuploidy in LO2 cells.

## Background

Chromosomal or genetic instability (CIN) leading to an aberrant chromosome number (aneuploidy) is a hallmark of cancers[1]. A growing body of evidence suggests that defects in the spindle checkpoint, a surveillance mechanism crucial for the proper segregation of chromosomes during every cell division, might promote aneuploidy and tumorigenesis [2]. The spindle checkpoint machinery consists of several proteins that are well-conserved in various species. These checkpoint proteins are recruited and activated at the kinetochores of unattached and/or unaligned chromosomes, and subsequently inhibit the anaphase-promoting complex/cyclosome (APC/C) and prevent the ubiquitination of substrates whose destruction is required for advance to anaphase [3]. To date, two checkpoint proteins are known for directly mediating the activation or/and inactivation of spindle checkpoint, i.e., CENP-E and BubR1 [4-6].

CENP-E is a kinesin-like motor protein localized on the kinetochore. It has an apparent molecular mass of 312 kDa, with an ATP-dependent motor domain located at the N-terminus. CENP-E is required for efficient capture and attachment of spindle microtubules by kinetochores, a necessary step in chromosome alignment during prometaphase [7-10]. Disrupting the function of CENP-E by various methods consistently results in the appearance of some unaligned chromosomes at metaphase. Previous studies using either microinjection or the antisense approach showed that cells with CENP-E defects had prolonged mitotic arrest, and even initiated apoptosis [11,12].

Hepatocellular carcinoma (HCC) is one of the most common carcinoma causing death world widely. However, genetic events in hepatic carcinogenesis are poorly understood. It has been reported that CIN can be observed in hepatoma carcinoma cell, resulting from defects of spindle checkpoint genes. Sze KM et al have shown that all 6 hepatoma cell lines with defective mitotic checkpoint showed significant reduced expression of mitotic arrest deficient 2 (*Mad*2)[13]. *Mad*1beta, a novel splicing variant of mitotic arrest deficient 1 (*Mad*1), was expressed at both mRNA and protein levels in the nine hepatoma cell lines tested and was over-expressed in 12 of 50 (24%) human HCC tissues[14]. Jeong SJ et al have shown that transcriptional dysfunction of *hsMad*2 is frequently observed in hepatocellular carcinoma cells [15]. Marchio et al used Comparative Genomic Hybridization (CGH) to evaluate and map genomic aberrations in 50 hepatocellular carcinomas from patients chronically infected with hepatitis B virus (HBV), and found nonrandom genomic imbalances and spindle checkpoint genes alterations [16]. Thus, the present study is designed to investigate the alteration of CENP-E gene expression in human hepatocarcinoma tissues, and study the fate of LO2 cells (normal liver cell line) treated with CENP-E shRNA vectors, with a intend to explore the role of CENP-E in human hepatocarcinogenesis.

## Methods

### Samples

Twenty-one HCC tissue samples and eighteen para-cancerous tissue samples were obtained from the Department of Surgery of the Liver & Biliary, the first and second affiliated hospitals of Chongqing Medical University, all of which were confirmed by pathobiology. Informed consents were obtained from all patients, and the medical ethical committee of Chongqing Medical University approved this study.

### Cell culture and transfection

LO_2 _and HepG2 cells were cultured in Eagle's Minimum Essential Medium media containing 100 mL/L fetal bovine serum. Transfections were carried out with shRNA vector and Lipofectamine 2000 transfection reagent (Invitrogen) mixture. These components were mixed in DMEM (serum free) according to the manufacturer's instructions. For mock transfections, cells were treated with Lipofectamine 2000 alone.

### Design of target sequence of shRNA

Specific oligonucleotide sequences used to knock out human CENP-E (NM_003870) were as follows: 1) 5'-CGCAGTCGTTCTCATACCAT-3', 2) 5'-CCACG GATGCTGGTGACCTC-3', 3) 5'-CGCACGGATGCTGGTGACCTC-3'. By BLAST analysis, these sequences have no homology with other coding sequences in human. Scrambled sequence used as negative control: 5'-CGAGTAAGACCATTCA GGTC-3'. The 5'end of this sequence corresponds to the cut-off point for *BamH*I enzyme (GATCC), while the 3'end, containing the T6 sequence, corresponds to the cutting site for *Hind *III enzyme (AGCTT). A ring sequence of 9 base pairs exists between the sense and anti-sense strands (TTCAAGAGA).

### Construction of shRNA expression plasmid

Two strands of oligonucleotides would undergo annealing, ligation, and transformation. To identify positive clones, the constructed shRNA expression plasmids were identified by sequencing in Takara Biotechnology Company. shRNA vectors were named pGenesil-CENPE 1, 2, 3 and pScramble (negative control) respectively.

### Real-time PCR analysis

Total RNA were isolated from adherent cells and clinical samples using the TRIZOL reagent (Invitrogen). First-strand cDNA was synthesized from 0.5 μg of total RNA by using random hexamers. The primers used for quantitating CENP-E mRNA were 5'-GCGATGGAAGAACAACTAGGTACC-3 '(forward) and 5'-GTTG CTTGGGACTGTAAAAGCTGT-3 ' (reverse) with a TaqMan-MGB(genecore, china) probe 5'(FAM)-AAAACGAGCACAGCGAAGAATAGCCAGAA-3'. Because CENP-E degradation kinetically follows the proteolysis of Cyclin B1 in anaphase, Cyclin B1 mRNA was used to normalize CENP-E mRNA, for which the primers and TaqMan-MGB probe were 5'-AGCACCTGGCTAAGAATG-3'(forward), 5'-CTTCGATGTGGCATACTTG-3'(reverse), and 5'(FAM) - ATCAAGGACTTACA AAGCACATG ACTGTC-3'. The PCR cycling program was 94°C for 5 minutes, then 40 cycles of 94°C for 30 seconds, 51°C for 30 seconds.

### Western Blotting

For CENP-E protein level analysis, cells and tissues were lysed with RIPA lysis Buffer, supplemented with protease inhibitors. The lysates were cleared by centrifugation at 14,000 rpm for 30 min at 4°C and quantitated by Bradford Protein Assay. Protein was enriched by immunoprecipitation method, and the precipitates were boiled with SDS-loading buffer, separated on 40-120 g/L and 100 g/L SDS-PAGE respectively, and then transferred onto polyvinylidene difluoride membrane (Millipore). Thereafter, the membrane was probed with affinity-purified mouse monoclonal antibody against human CENP-E (Abcam, USA) and mouse monoclonal antibody of Cyclin B1(Abcam, USA), followed by horseradish peroxidase-conjugated secondary antibody. After washing, the membrane was incubated in ECL Plus reagent before detection. Then, the blots were scanned in grey scale and analyzed using QUANTITY ONE software.

### Immunofluorescence Microscopy

LO_2 _cells were seeded onto sterile, acid-treated 12-mm coverslips in 24-well plates. Nocodazole-treated LO_2 _cells were applied to poly-L-lysine-coated coverslips. The coverslips were rinsed in PBS, and the cells were fixed by 4% paraformaldehyde for 10 min plus 2 ml/L Triton X-100 for 10 min at room temperature. The cells were then incubated at 37°C sequentially with: (a) mouse anti-CENP-E monoclonal antibody (1:250;Abcam), (b) Rhodamine-conjugated goat anti-mouse IgG (1:20, KPL), and (c) 0.1 μg/ml 4',6'-diamidino-2-phenyl-indole (DAPI). Cells were rinsed extensively in PBS between each incubation, and all reagents were diluted in PBS/5% bovine serum albumin. Finally, the coverslips were mounted and viewed in a confocal microscopy (SP5, Lecia). All images in each experiment were collected on the same day using identical exposure times.

### MTT assay

For measurements of cell proliferation rates, cells were planted into 96-well plates at a density of 1 × 10^3^/100 μl. Then, the plates were incubated for 1, 2, 3, 4, 5, 6 or 7 days, added into MTT solution (10 μl/well), incubated for 4 h at 37°C, and measured the absorbance of 450 nm UV in a microplate reader. Each assay was done in triplicate wells, and each experiment was repeated three times.

### Measurement of apoptosis

After 24 hours of transfection, digested the cells of each group by Trypsin, suspended them in PBS, and centrifuged them for 10 min at 1000 rpm. Then, discarded the supernatant, resuspended the pellet cells in 500 μl of 1× Binding Buffer into which added 5 μl annexin V-PE staining solution, and incubated them at room temperature for 5 min in the dark.

### Chromosome counts

After treated with nocodazol (Sigma-Adrich) for 3 hours, the cells were incubated 6 hours,, centrifuged 5 minutes at 2500 rpm, and resuspended in 5 ml hypotonic solution (0.05 M KC1: 0.25% trypsin EDTA, 3:1) and maintained at 37°C for 20 minutes. Then 1 ml fixative (methanol:acetic acid, 3:1) was added into the tube, and the suspension was centrifuged immediately. The pellet was resuspended in 5 ml of methanol for 5 min, and then the cells were centrifuged and resuspended in 5 ml fixative. This step was repeated twice. After centrifugation, the cell pellet was dropped onto chilled wet slides and immediately put under a hot air flow to evaporate the fixative rapidly.

### Statistical analysis

The SPSS 13.0 software was used to establish database for statistical analysis. The data were represented in form of  ± *s*. Single-factor variance analysis and Independent-Samples T Test were used, where *p *value less than 0.05 was considered as statistical significance.

## Results

### Reduced expression of CENP-E in HCC tissues and HepG2 cells

**Figure 1:**
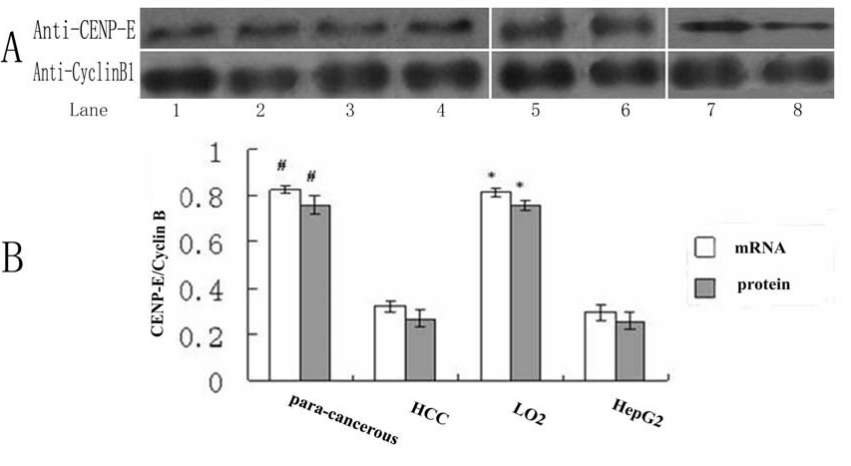
**shows that CENP-E expression in HCC and para-cancerous tissues, LO2 and HepG2 cell lines**. (a) Analysis of CENP-E protein levels by Western blot. lysis extracts derived from para-cancerous tissues (lane 5-6), HCC tissues (lane1-4), LO2 (lane 7) and HepG2 cell lines (lane 8), Cyclin B1 was simultaneously immunoprobed for loading control. (b) QPCR and western blot analysis for CENP-E of tissues and cell lines, Cyclin B1 serves as loading control. Data represent the mean ± S.E. of three independent experiments. ^#^, *P *< 0.05 versus HCC tissues; *, *P *< 0.05 versus HepG2 cells

Real-time quantitative PCR (QPCR) and western blot analysis were used to characterize the expression of CENP-E in HCC and para-cancerous tissues, and HepG2 and LO_2 _cells. The level of CENP-E was normalized by Cyclin B1. Results showed that the mRNA level of para-cancerous tissue (0.826 ± 0.014) was significantly higher than that of HCC tissue (0.321 ± 0.023)(t = 12.1, *P *= 0<0.0). To confirm the results from clinical tissues, we investigate the level of CENP-E mRNA in HepG2 and LO_2 _cells. After treating them with nocodazole, we collected the mitosis cells for detection, and found that the level of CENP-E mRNA in LO_2 _cells (0.814 ± 0.019) was significantly higher than that of HepG2 cells (0.239 ± 0.019)(t = 17.9, *P *= 0<0.05)(fig. 1B).

The results of western blotting were consistent with those of QPCR, CENP-E protein level in HCC tissues (0.267 ± 0.038), as measured by western blot, were diminished by about one-fold as compared with that of the para-cancerous tissues (0.762 ± 0.041)(t = 12.2, *P *= 0<0.05), and only about half of CENP-E in HepG2 cells (0.257 ± 0.039) extract could be detected as compared in LO_2 _cell extract (0.759 ± 0.023) (fig. 1A) (t = 13.2, *P *= 0<0.05).

### Transfection with CENP-E shRNA efficiently knocked down CENP-E in the LO_2 _Cells

**Figure 2:**
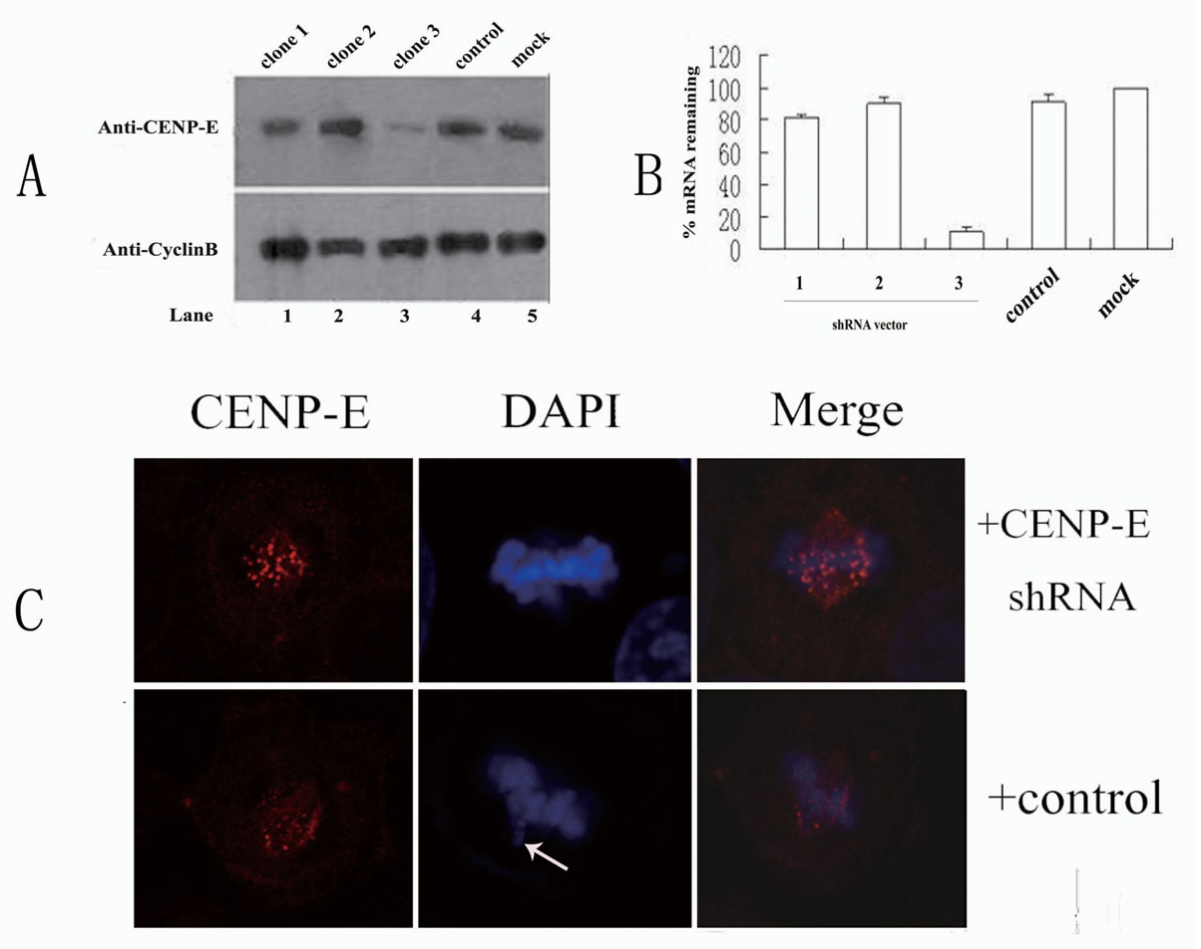
**Analysis interferer efficiency of pGenesil-CENPE**. (A)Analysis of CENP-E protein levels by Western blot. Seventy-five micrograms of mitotic extracts derived from LO2 cells treated by nocodazol before detection for 3 h (lane 1-5). (B)shRNA-induced reduction of CENP-E mRNA and protein levels. Reduction of CENP-E mRNA. LO2 cells were transfected with various CENP-E shRNA vectors as indicated, and the mRNA levels were measured 24 h posttransfection by QPCR. Control:negative control (pScramble); mock: transfected Lipofectamine 2000 only alone. (C)Immunofluorescence of CENP-E of LO2 cells 24 h posttransfection with control shRNA vector or CENP-E siRNA. Cells were double stained with DAPI (4,6-diamidino-2-phenylindole) and CENP-E antibodies. Identical exposure times were used for imaging both control and CENP-E shRNA-transfected cells (white arrow point to misaligned chromosome). Bar, 5 μm.

shRNA vector targeting for CENP-E and control shRNA vector were delivered into LO_2 _cells, and their knockdown efficiencies in LO_2 _cells were compared. QPCR analysis consistently showed an 75~80% reduction of CENP-E mRNA 24 h after transfection of cells with clone 3, which was used for the remaining of this study (Fig. 2B). Next, we examined the knockdown of CENP-E at the protein level by Western blotting. We compared the level of CENP-E protein in extract of cells 24 h after transfection with pGenesil-CENPE3 with those untransfected cells and transfected with pScramble. Only a small amount of CENP-E was detected in 75 mg of lysates of pGenesil-CENPE3 transfected cells. CENP-E protein levels, as measured by quantitative immunoblotting, were diminished by at least 7-8 fold as compared with those untransfected cells and pScramble transfected cells (Fig. 2A, top). Meanwhile, we detected the amount of CENP-E protein at single cell level by indirect immunofluorescence assay. In pScramble-transfected cells, the signals corresponding to CENP-E were readily detected in mitotic cells (Fig. 2C, top); however, in CENP-E shRNA-transfected cells, signal was undetectable. Therefore, the shRNA vector can efficiently knockdown the CENP-E in LO2 cells.

### Deletion of CENP-E induced apoptosis and slowed down proliferation in LO_2 _cells

**Figure 3:**
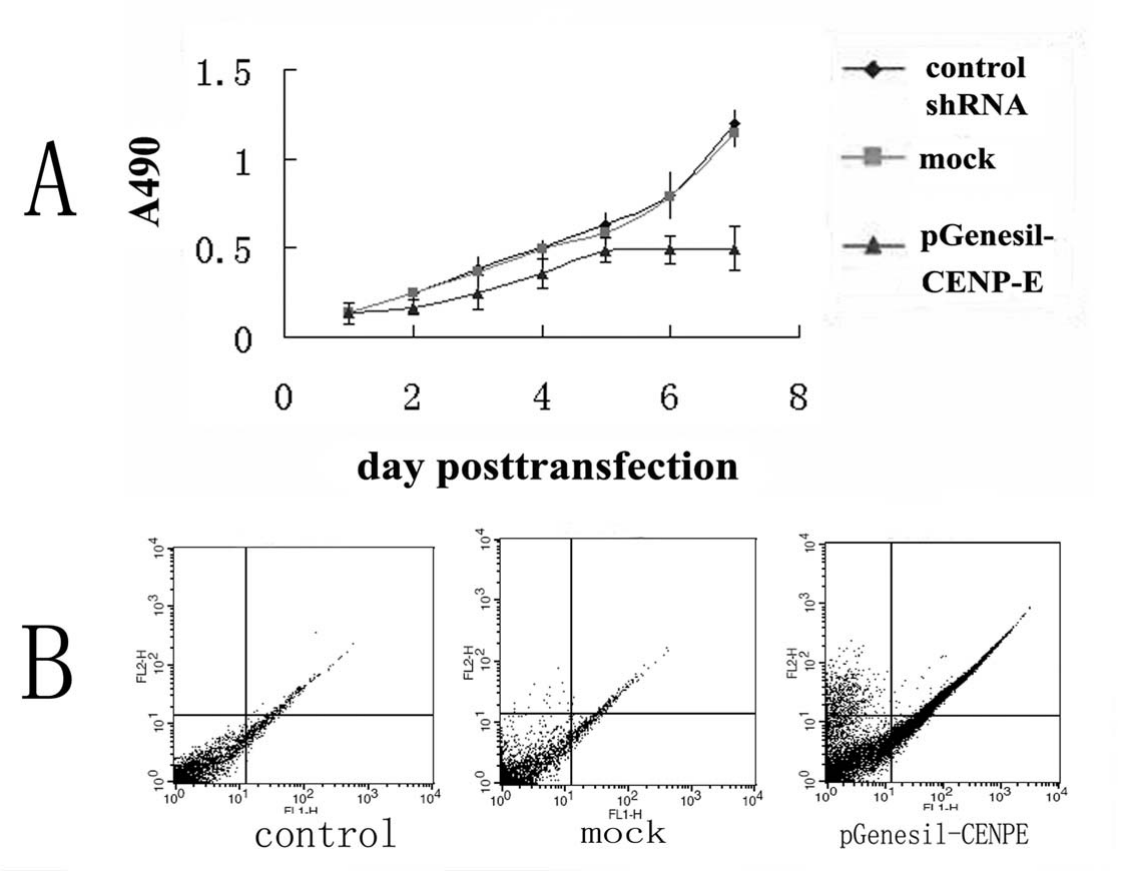
**proliferation and apoptosis analysis by MTT assay and flow cytomerty**. (A) shows that proliferation of LO2 cells expression shRNA. Proliferation of shRNA-transfected LO2 cells (clone 3), shRNA scramble control and un-transfected LO2 cells were analyzed by MTT assay as described earlier. The mean ± SE of three independent experiments are shown. LO2 cells transfeced with pGenesil-CENPE vector proliferation slowed. (B) the result of flow cytometry showed that the percent of apoptosis cells of LO2 cells transfected with pGenesil-CENPE vector is higher than cells transfected with scrambler control shRNA vector or mock.

Cell proliferation activity was examined using MTT assay. The proliferation rate of pGenesil-CENPE3-transfected cells was lower than that of pScramble-transfected and untransfected LO_2 _cells (fig. 3A). The percentage of apoptosis [(16.57 ± 1.4)%] in pGenesil -CENPE3 mediated cells was significantly higher than that in cells transfected with pScramble [(2.84 ± 0.84)%] (t = 29, P = 0<0.05) and mock vectors [(2.61 ± 0.4)%] (t = 33, P = 0<0.05). Apoptosis in cells transfected with pGenesil-CENPE3 was presented in fig. 3B.

### Depletion of CENP-E caused aneuploidy in LO_2 _cells

**Figure 4:**
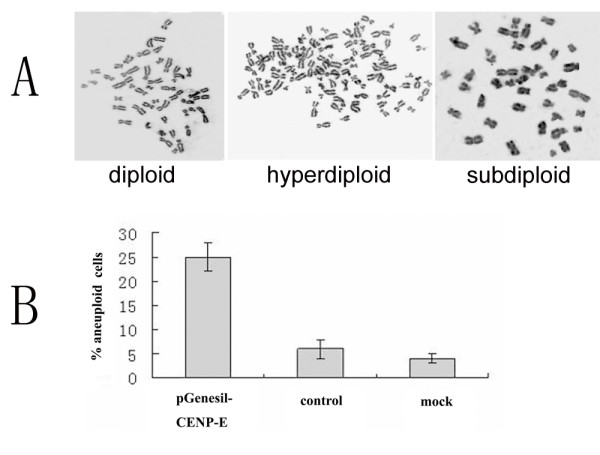
**Effect of pGenesil-CENP-E on chromosome sepration in LO_2 _cells**. (A) shows that karyotype of LO2 cells, tetraploid (middle) and subdiploid karyotype (right) present in pGenesil-CENPE mediated LO_2 _cells. (fig 4A). (B) aneuploid cells in the LO2 cells treated with shRNA vector is largely high, compare with cells transfected with scrambler control shRNA vector or mock. Data represent the mean ± S.E. of three independent experiments. *, *P *< 0.05 versus mock; ^#^, *P *> 0.05 versus mock; (fig 4B)

To investigate whether depletion of CENP-E in LO2 cells affected the separation of chromosome and cause aneuploid cells, cells transfected with pGenesil-CENPE3 and pScramble were analyzed by chromosome account 24 h later (fig. 4A). Results demonstrated that aneuploid increased significantly in pGenesil-CENPE3-treated LO2 cells [(25.1 ± 2.8)%], compared with those in pScramble-treated [(5.57 ± 1.8)%] (t = 44.2, P = 0<0.05) and untrasfected cells [(4.69 ± 1.3)%] (t = 50.9, P = 0<0.05) (fig. 4B).

## Discussion

The centromere proteins are crucial for centromere assembly and centromere function. CENPs dysregulation have been reported in some cancers tissues or cell lines. Centromere protein-A overexpress in human primary colorectal cancer and HCC [17,18]. Obrien et al [19] found that the expression of centromere protein CENP-F was associated with the prognosis of early breast cancer. CENP-H expression was higher in tongue cancer cell lines and nasopharyngeal carcinoma cell lines [20,21]Therefore, to study centromere proteins may contributes to exploring the mechanism of chromosome segregation, revealing the mechanism of malignant cellular proliferation and finding cancer marker proteins, and also may provide new targets for carcinoma therapy and prognosis estimation of cancer patients.

### Reduced expression of CENP-E in human hepatocellular carcinoma

CENP-E is also one of the components directly responsible for capturing and stabilizing spindle microtubules by kinetochores [9,10]. CENP-E interacts with BubR1 and stimulates its kinase activity, which implicates its role in activating and maintaining mitotic checkpoint signalling [6,19]. Deletion CENP-E by various methods could impair the function of spindle checkpoint [9,12]. In this study we found that the mRNA and protein expression levels of CENP-E were reduced both in HCC tissues and in human hepatocellular carcinoma-derived cell lines (HepG2), and that the LO_2 _cells transfected with shRNA vector had a decreased proliferation rate and an increased proportion of aneuploid and apoptosis cells.

### Reduced expression of CENP-E may be involved in human hepatocarcinogenesis

Our evidence presents that the level of CENP-E protein was reduced in the HCC tissues, which implicates that CENP-E may be involved in human hepatocarcinogenesis. We draw this conclusion from two aspects as follows:

(1) Aneuploidy is related with tumorigenesis. A majority of human cancer cells are aneuploid due to an underlying chromosomal instability phenotype [22]. Theodor Boveri proposed an aneuploid hypothesis, in which, aneuploid was presumed as a direct cause of cancerous transformation [23]. With the discovery of oncogenes and tumour suppressors in the late 1970s and 1980s, some researchers suggested that heterozygosity loss might result in the phenotypic expression of mutated tumour suppressor genes in the aneuploid cell, and aneuploid cells may show chromosome polysomy that harbours oncogenes [24]. Aneuploid is still an important cause of tumorigenesis, and oncogenes hypothesis also supports this point, although there is no direct evidence to confirm that aneuploidy is a primary contributor to tumorigenesis up to now.

(2) Cancer is associated with weakened spindle checkpoint. A growing body of evidence suggests that defects in the spindle checkpoint might promote aneuploidy and tumorigenesis. Mouse with reduced expression of spindle checkpoint proteins survived but developed aneuploidy at an elevated rate, and in some, but not all cases, these animals are more susceptible to spontaneous tumours [25,26] Cells over-expressing Mad2 developed a large number of chromosome breaks, fragments, and fusions in addition to whole chromosomal aneuploidy [27]. Mutation of mitotic checkpoint genes such as BubR1, Mad2, has been found in colon cancer lines, and breast cancer, respectively [28,29]. Mosaic variegated aneuploidy (MVA), which is characterized by an increase in aneuploidy (>25% of cells exhibit near-diploid aneuploidy) and childhood cancers [30]. Five of eight MVA patients were found to have mutations in both alleles of BubR1 gene. Aneuploidy occurred in the pGenesil-CENPE shRNA-treated LO_2 _cells in this study, for which one potential explain is that the level of CENP-E may affect spindle checkpoint. Once the level of CENP-E protein was decreased, the onset of unaligned chromosomes and aneuploidy was induced in the anaphase. Completely inactivating the checkpoint would result in cell autonomous lethality because of large loss or gain of chromosome; however, cells with a weakened checkpoint could survive but exhibit chromosomal instability. In our study, the level of CENP-E protein was down-regulated dramatically, thus the spindle checkpoint of LO_2 _cells treated with shRNA vector might be subjected to a large degree of damage, some of which even suffer apoptosis or death. These points are also proved by our MTT result and are consistent with those of Marcel Tanudji [31].

### The controversy about the role of reduced CENP-E in hepatocarcinogenesis

Beth A.A. Weaver has demonstrated that aneuploidy resulted from CENP-E_+/-_, which acts as an oncogene as well as a tumour suppressor. Widespread aneuploidy was accompanied by a 50% decrease of spontaneous liver tumours in aged CENP-E_+/- _mice compare with CENP-E_+/+ _mice [32]. In the present study, we found that CENP-E decreased by about 50% in HCC tissue as compared with that in para-cancerous tissue. Possible explanations for these contradictions may be:

(1) Firstly, We tentatively put forward that the threshold level of CENP-E protein for promoting tumorigenesis might be in the range of 20-50% of the normal. The rate of apoptosis or death increased obviously in LO_2 _cells, when CENP-E was down-regulated to 15-20% in this study. However, aneuploidy due to reduced CENP-E (about 50% of the normal level) in CENP-E+/- mouse could act as a tumour suppressor. CENP-E in HCC tissue may be lower than the threshold value and higher than 15-20% of the normal level, and then may be promoting hepatocarcinogenesis.

(2) Secondly, the control samples used in our study may affect our final results. Because the expression level of CENP-E protein in para-cancerous may be lower than that of the normal liver tissue which was unavailable in the present study, the level of CENP-E in HCC tissue may be no higher than 50% of the normal.

(3) Finally, our results supported the following hypothesis, as proposed previously by Salmon's and Yen's laboratories [33]. A certain level of the waiting-anaphase signal may be required for cells to induce mitotic arrest. When CENP-E is reduced lightly in cells, the accumulation of the signals generated at each kinetochore is still sufficient to reach the threshold required for mitotic arrest, and anaphase onset accompanied unaligned chromosomes promoting aneuploidy. When CENP-E is reduced to a larger extent, the accumulation of the signals may not be sufficient to arrest mitosis, and cells possessing mitosis with large loss or gain of chromosome may suffer apoptosis or death.

Despite the fact that reduced expression of CENP-E protein was found in HCC tissues and could induced apoptosis and aneuploidy in LO2 cells, our results do not provide direct evidence that reduced expression of CENP-E can initiate hepatocarcinogenesis. However, this problem might be solved if we down-regulate the level of CENP-E to various degrees by constructing interfere vector or finding microRNA to target CENP-E, and investigate the relationship between the reduced CENP-E expression and hepatocarcinogenesis.

In a word, we found that CENP-E expression was reduced in HCC tissue, and reduced CENP-E expression could interfere with the separation of chromosome in LO2 cells.

## Conclusions

Together with other results, these results reveal that CENP-E expression was reduced in human HCC tissue, and low CENP-E expression result in aneuploidy in LO2 cells.

## Competing interests

The authors declare that they have no competing interests.

## Authors' contributions

ZL participated in study design, carried out most of the experiments, and drafted the manuscript. KL participated collecting samples, and participated in manuscript preparation. XW participated in study design and revised the manuscript. JC participated in study design and revised the manuscript. BL participated in the critical revision of the manuscript. SL participated in the molecular genetic studies. QS participated in the Statistical analysis. YC participated in the critical revision of the manuscript. CY participated in the collecting tissues from hospital and samples prepare. YZ participated in cell culture. YW conceived of the study, participated in its design and coordination. All authors read and approved the final manuscript.
